# Vaccination with Bivalent DNA Vaccine of α1-Giardin and CWP2 Delivered by Attenuated *Salmonella typhimurium* Reduces Trophozoites and Cysts in the Feces of Mice Infected with *Giardia lamblia*

**DOI:** 10.1371/journal.pone.0157872

**Published:** 2016-06-22

**Authors:** Xian-Min Feng, Wen-Yu Zheng, Hong-Mei Zhang, Wen-Yan Shi, Yao Li, Bai-Ji Cui, Hui-Yan Wang

**Affiliations:** 1 The Department of Pathogenic Biology, Jilin Medical University, Jilin City, China; 2 The Center Hospital of Jilin City, Jilin City, China; University of South Dakota, UNITED STATES

## Abstract

**Background:**

*Giardia lamblia* is one of the most common infectious protozoans in human that may cause diarrhea in travelers. Searching for antigens that induced effectively protective immunity has become a key point in the development of vaccine against giardiasis.

**Methodology/Principal Findings:**

Mice vaccinated with *G*. *lamblia* trophozozite-specific α1-giardin DNA vaccine delivered orally by attenuated *Salmonella typhimurium* SL7027 elicited 74.2% trophozoite reduction, but only 28% reduction in cyst shedding compared with PBS buffer control. Oral vaccination with *Salmonella*-delivered cyst-specific CWP2 DNA produced 89% reduction in cysts shedding in feces of vaccinated mice. Significantly, the mice vaccinated with *Salmonella*-delivered bivalent α1-giardin and CWP2 DNA vaccines produced significant reduction in both trophozoite (79%) and cyst (93%) in feces of vaccinated mice. This parasite reduction is associated with the strong local mucosal IgA secretion and the IgG2a-dominant systemic immune responses in vaccinated mice.

**Conclusions:**

The results demonstrate that bivalent vaccines targeting α1-giardin and CWP2 can protect mice against the colonization of *Giardia* trophozoite and block the transformation of cyst in host at the same time, and can be used to prevent *Giardia* infection and block the transmission of giardiasis.

## Introduction

*Giardia lamblia* is a protozoan parasite that cause diarrhea in travelers[1].The prevalence of giardiasis has been reported to be as high as 20–40% in developing countries [2]. People get infected by the ingestion of cysts in contaminated water or food. *Giardia* is highly contagious, since ingestion of as few as ten cysts can cause infection [3]. In small intestine, ingested cyst releases trophozoite that causes giardiasis with symptoms of diarrhea, abdominal pain, malabsorption and weight loss. Trophozoites are transformed into cysts in the gut lumen. Cysts contain the hard cyst wall which enables to resist the harsh environmental conditions when the cysts are passed out in the feces [4].

To date, the preventive medical strategies for giaridiasis are not available despite the clinical importance of *G*. *lamblia*. A crude veterinary *Giardia* vaccine called GiardiaVax®, composed of total lysate of *G*. *lamblia* trophozoites, relieved giardiasis symptoms and reduced cyst shedding in the feces of vaccinated cats and dogs [5]. There is no vaccine with success in eliciting sustainable and effective protective immunity in human due to the complex antigen composition and antigen variation. Searching for antigens that induced effectively protective immunity has become a key point in the development of vaccine against giardiasis.

The best-characterized *Giardia* antigens are members of the family of variable surface proteins (VSPs), which constitute a major fraction of surface proteins in trophozoites [6]. However, the high variability in antigenicity and cross reactivity with other antigens suggest that VSPs are not promising targets for the development of vaccine against *Giardia* infection [7–10].

Encystation and excystation are two key events in *Giardia* life cycle. The survival of *Giardia* trophotoites in the host and the discharge of cyst are two important developmental stages during infection, pathogenesis and transmission of *Giardia*. So a more effective vaccine to control the giardiasis should block both encystation and excystation of the parasite. The ideal vaccine targets should be particularly critical in the transformation stage between cyst and trophozoite.

The cyst wall proteins (CWP) are the important components of cyst wall and their expression is cyst-specific and necessary for the transformation from trophozoite stage to cyst stage [11]. Especially, the cyst wall protein-2 (CWP2) plays a pivotal role in the process of *Giardia* excystation [12]. Several live and DNA vaccines against CWP2, blocked switching of *Giardia* from trophozoites to cysts and reduced cyst shedding [13–15]. The transmission-blocking vaccine induced reduction of cyst shedding attributes to the specific antibodies that target CWP2 and inhibit the formation of the cyst structure. No differences in the trophozoite numbers in the small intestine of mice immunized with CWP2 was observed compared to non-immunized group, which limits its potential clinical utility [14].

The α-giardins are trophozoite-specific proteins which belong to an annexin-related big family. They are expressed mainly in trophozoite and released from phospholipid membrane when calcium concentration is high. The released α-giardins are very immunogenic during acute giardiasis and induce strong antibody response during infection [16, 17].The α1-giardin-based vaccine can induce Th1-type immune responses and systemic IgG2a that may be critical for the effective immune protection against *Giardia* infection [18]. The expression of α1-giardin in excysting cells with transformation of cyst to trophozoites which cause disease [16, 19], and its shedding in the feces [19] indicate α1-giardin plays important role in the infection and pathogenesis. So far, the evidence has not shown that the α1-giardin-based vaccine can block the encystation of *Giardia*. An ideal vaccine against giardiasis not only protects against infection but also blocks the disease transmission. *Giardia* trophozoites and cysts express quite different antigens [4, 20]. So single antigen from trophozoite or cyst may not protect against infection and block the transmission at the same time. The bivalent vaccine containing both trophozoite-specific α1-giardin and cyst-specific CWP2 may play these roles.

*G*. *lamblia* is a parasitic protozoan dwelling in the small intestine of human and mammal animals and causes giardiasis [21]. Due to the importance of gut-associated lymphoid tissue (GALT) in the immune response to enteric pathogens, a ideal vaccine against gastro-intestinal pathogens should be able to access GALT through mucosal M cells located predominantly in the Peyer’s patches (PP) and therefore activate mucosal defenses in the intestinal tract [22–24]. *Salmonella* usually infects the host through invading the epithelial barrier of the gut by M-cell active uptake [23, 24], then reaches to the mesenteric lymph nodes (MLN) through GALT, some of them gets directly into the blood circulation by uptaking into macrophages, and dendritic cells (DCs) [25]. For these reasons, the *Salmonella* is a natural adjuvant by the presence of pathogen-associated molecular patterns. *Salmonella*-based vaccine systems are among the most advanced and promising technologies for inducing immunological protection against enteric pathogens [18, 26, 27]. The attenuated *S*. *typhimurium* SL7207 strain with the pathogenic aroA gene deleted doesn’t cause disease in inoculated mice therefore is a safe vector for delivering vaccine [28].

In this study, we combined the trophozoite-specific α1-giardin and cyst-specific CWP2 antigens to develop a novel *Salmonella*-based oral bivalent vaccine against giardiasis. We show here that the co-vaccination of this bivalent vaccine significantly reduces both trophozoites and cysts in feces of vaccinated mice after being challenged with *G*. *lamblia* trophozoites.

## Methods

### *G*. *lamblia* culture

*G*. *lamblia* isolate C2 was derived from a patient in Southwest China [29] and identified as Genotype A [30]. The trophozoites of this isolate were axenically cultured in modified TYI-S-33 medium, pH 7.0, supplemented with 10% heat-inactivated bovine serum (Hangzhou Sijiqing Biological Engineering Materials, Hangzhou, China) and 0.05% bovine bile (Sigma, US) in borosilicate glass screw-cap culture tubes without shaking at 37°C as described previously [29]. The cultures were subcultured with initiating 2×10^5^ trophozoites per 4ml tube three times a week to expand the parasites. The total culture were chilled on ice for 20 min to detach trophozoites from the tube wall and trophozoites collected by centrifuging at 2,000 rpm at room temperature for 10 min followed by a washing step with 1×PBS and counted.

### The expression of recombinant CWP2-S (soluble fragment of CWP2) and α1-giardin proteins and production of antisera

The cDNA encoding for C-terminal 125 amino acids soluble fragment of *Giardia* CWP2 (GenBank accession No: XM_001710190.1) (CWP2-S) was codon optimized based on bacterial codon preference and synthesized by BGI (Shengzhen, China). The synthesized DNA was cloned into bacterial expression vector pET30a (Novagen) using BamHI and XhoI sites. The DNA encoding for the full-length α1-giardin (Genbank accession No: XM_001704258.1) was amplified from the total cDNA of *G*. *lamblia* C2 isolate using primer sets (5’-TCCATATGCCGAAGGTCACCGACAT-3’, 5’-TACTCGAGCTTCACGCGCCAGAGGGTGC-3’) and subcloned into pET41a using NdeI and XhoI sites with fusion GST. The recombinant CWP2-S (rCWP2-S) and α1-giardin (rα1-giardin) proteins with 6 histadine-tag at N-terminus (CWP2-S) or at C-terminus (α1-giardin) were expressed in *E*.*coli* BL21 under 1mM IPTG induction. The recombinant proteins with His-tag were purified using Ni-affinity chromatography according the manufacturer’s instructions (GE Healthcare,Sweden). The rabbit antisera were made by immunizing each two female New Zealand white rabbits with age of 6 months provided by the Animal facility at Jilin University, each hosted in a cage with 60 x 60 x 40 cm, with 200 ug of rCWP-S or rα1-giardin formulated with Freud’s complete adjuvant (FCA) and boosted 3 weeks after with the same amount of proteins formulated with Freud’s incomplete adjuvant (FIA). Two weeks after the boost immunization, the rabbits were anaesthetized with 30 mg/kg of Ketamine intramuscularly, then euthanized with exsanguination. The rabbit sera were isolated from collected blood. The anti-α1-giardin and CWP-S IgG titers were measured in the collected sera using indirect enzyme-linked immunosorbent assay (ELISA).

### Bacterial strain

The attenuated *S*. *typhimurium* SL7207 strain with the pathogenic aroA gene deleted [28] was kindly provided by Prof. She Feifei (Fujian medical university) and cultured in LB broth.

### Construction and transformation of the plasmid pVAX1-α1-giardin and pVAX1-CWP2

The DNAs encoding for full-length α1-giardin and CWP2 (without signal peptide) were amplified from *G*. *lamblia* total cDNA using the primer sets R3 (5’-CCAAGCTTATGCCGAAGGTCACCGACAT-3’/ 5’-CGGGATCCCTTCAC GCGCCAGAGGGTGC-3’) and R4 (5’-CCAAGCTTATG GCCACCGAGGAGGA GGC-3’/5’-CGGGATCCCCTTCTGCGGACAATAGGCTT-3’), respectively. The amplified DNAs were cloned into the eukaryotic expression vector pVAX1 (Invitrogen, Carlsbad, USA) using HindIII/BamHI sites (pVAX1-α1-giardin and pVAX1-CWP2). The correct inserts and reading frames were confirmed by double stranded DNA sequencing (Shenggong, Shanghai, China). The coding sequences for α1-giardin and CWP2 amplified from C2 isolate are identical to the sequences from GenBank mentioned above.The recombinant plasmid DNAs, pVAX1-α1-giardin and pVAX1-CWP2, as well as the plain vector pVAX1 were transformed into attenuated *S*. *typhimurium* strain SL7207 using electroporation as described previously [31]. The positive transformants were selected on LB agar containing 50 μg/ml kanamycin and further identified by PCR amplification using gene-specific primers.

### Stability testing of the plasmid pVAX1-α1-giardin and pVAX1-CWP2 in SL7207

To determine the maintenance of the recombinant plasmids in the transformed *S*. *typhimurium* SL7207, the transformed bacteria were cultured for up to 7 days without antibiotic selection. The stability of the plasmid maintained in the bacteria was determined by comparing the number of survived bacteria on plate containing kanamycin (containing the plasmid) to the total count on plate without kanamycin at each 12 h interval. Four colonies were randomly picked up from each bacteria transformed with pVAX1-α1-giardin and pVAX1-CWP2 (without kanamycin selection) to extract plasmid DNAs. The extracted plasmid DNAs were used for PCR amplification with gene-specific primers and digested with HindIII and BamHI to release the insert as a confirmation of the correct inserts in the extracted plasmid DNAs.

### Mouse immunization and challenge with G. lamblia trophozites

Female BALB/c mice aged 6–8 weeks and free of specific pathogens were provided by the Animal facility at Jilin University, China. All mice were kept in cages with each cage hosted less than 5 mice in animal facility with room temperature set at 22± 2°. The light cycle is 12 light/12 dark about 6 a.m. to 6 p.m. The room is well ventilated. The temperature, airflow and light timer functions are monitored daily. The mice were fed with adequate rodent diet food provided by Jilin University Animal facility and filtered water. After one week quarantine, the mice were divided into five groups with 30 animals each. Each group of mice was inoculated orally with 1×10^5^ bacteria of SL7207/pVAX1-α1-giardin, SL7207/pVAX1-CWP2-S, both SL7207/pVAX1-α1-giardin and SL7207/pVAX1-CWP2-S (each 1×10^5^), SL7207/pVAX1 or PBS control in a total volume of 100 μl, respectively. All mice were fasted and only fed with water containing 1% sodium bicarbonate to neutralize stomach acidity overnight before inoculation. Mice were boosted with the same dose of bacteria 8 days after the first inoculation. Seven weeks after the boost, 10 mice from each group were euthanized by CO2 inhalation plus cervical dislocation to obtain blood samples, intestinal lavage fluid (ILF) and mesentery lymph nodes for immunological tests as described below. The left 20 mice from each group were orally challenged with 1×10^5^ trophozoites of *G*. *lamblia* C2 isolate in 100 μl TYI-S-33 medium. Four days after infection, ten mice from each group were sacrificed. The small intestine was removed and opened in 10 ml of ice-cold PBS for 10 min, and washed vigorously with 50 ml ice-cold PBS to detach trophozoites. The washed liquid was centrifuged at 800×g for 10 min and the pellets were suspended in 1 ml PBS. The *Giardia* trophozoites were counted under a phase-contrast microscope. Other ten infected mice from each group were maintained for collecting feces daily for consecutive 2 weeks. The cysts shed in the feces were count under microscope and the mean number of cyst per gram feces was calculated.

During the mouse experiments, animals were monitored by research personnel daily for general appearance and any sign of disease. If any animal appears bleeding diarrhea, labored breathing, severe leg injuries or have become moribund it will be euthanized immediately by CO2 inhalation (mouse) to terminate the experiment. All mice tolerated well to the vaccination and challenge. No mouse died prior to the experimental endpoint.

### Evaluation of serological immune responses

The level of antigen-specific IgG in the sera was determined by standard ELISA. The 96-well microtiter ELISA plates (JET Biofil,China) were coated with rα1-giardin or rCWP2-S (1μg/well) overnight at 4°C. Plates were washed 3 times with PBS containing 0.05% Tween 20 (PBST) and then blocked with 5% bovine serum albumin (Sigma, US) in PBS for 2 h at 37°C. The mouse serum samples were serially diluted from 1:500 to 1:25,000 in PBST and added into the wells (in triplicate) and incubated for 1 h at 37°C. The plates were washed three times with PBST and incubated with 1:5,000 goat anti-mouse IgG conjugated to horseradish peroxidase (Bioss, China), and developed with 100 μL of 1 mg/mL 2,2’-azino-bis(3-ethylbenzthiazoline-6-sulfonic acid) (Sigma, US) and 0.003% hydrogen peroxide. Total IgG2a and IgG1 in serum samples were determined with an antibody isotyping kit for mouse (Thermo, US) following the manufacturer’s instruction. The absorption was determined at 450 nm using a microplate absorbance reader (Bio-Rad 680, USA).

### Measurement of the total secretory IgA (SIgA) and antigen-specific SIgA in ILF

To collect intestinal lavage fluid (ILF) for testing IgA antibody, about 10 cm of small intestine was excised from each mouse beginning from the gastroduodenal junction, flushed with cold PBS in total volume of 5 ml. The flushing liquids was collected by centrifugating at 3500×g for 15 min for measuring secretory IgA (SIgA) [13]. Total SIgA was detected using double antibody sandwich ELISA based on the manufacturer’s instructions (IBL, German). The antigen specific SIgA was measured by standard ELISA using α1-giardin or CWP2 coated plate. The Goat anti-mouse IgA conjungated with HRP (SAB, USA) was used as the second antibody. The absorption was determined at 450 nm using a microplate absorbance reader (Bio-Rad 680, USA).

### Detection of the α1-giardin and CWP2 expression in mesenteric lymph nodes (MLN)

The MLN collected from immunized mouse were fixed with 4% formaldehyde, embedded with paraffin and cryosectioned. The MLN sections were blocked with 5% bovine serum albumin (BSA) (DingGuo,China) for 1 hour at room temperature, then incubated with rabbit anti-α1-giardin sera or rabbit anti-CWP2 sera at 1:500 dilution in PBS containing 5% BSA overnight at 4°C. After being washed with PBST, the sections were incubated with 1:500 goat anti-rabbit IgG conjugated with DyLightTM 488 (DingGuo, China) for 1 hour at room temperature. The normal rabbit serum was used as negative control. The fluorescence reactions of sections were photographed under a fluorescence microscope (Leica, Germany).

### Data analysis

The statistical differences between groups were analyzed by one-way ANOVA using SPSS 11.5 software with *p* < 0.05 was considered as statistically significant and *p* <0.01 as highly significant. All values shown for each group are the mean ± SE.

### Ethics statement

All experimental animals were purchased from Animal Facility of Jilin University (Jilin, China). All experimental procedures were reviewed and approved by the Jilin Medical University Animal Care and Use Ethic Committee (*Permit Number*:JMU-0201) before we started the experiments and were consistent with the NIH Guidelines for the Care and Use of Laboratory Animals. All surgery was performed under sodium pentobarbital anesthesia, and all efforts were made to minimize suffering.

## Results

### Construction and stability in S. typhimurium of the plasmid pVAX1-α1-giardin and pVAX1-CWP2-S

**Fig 1:**
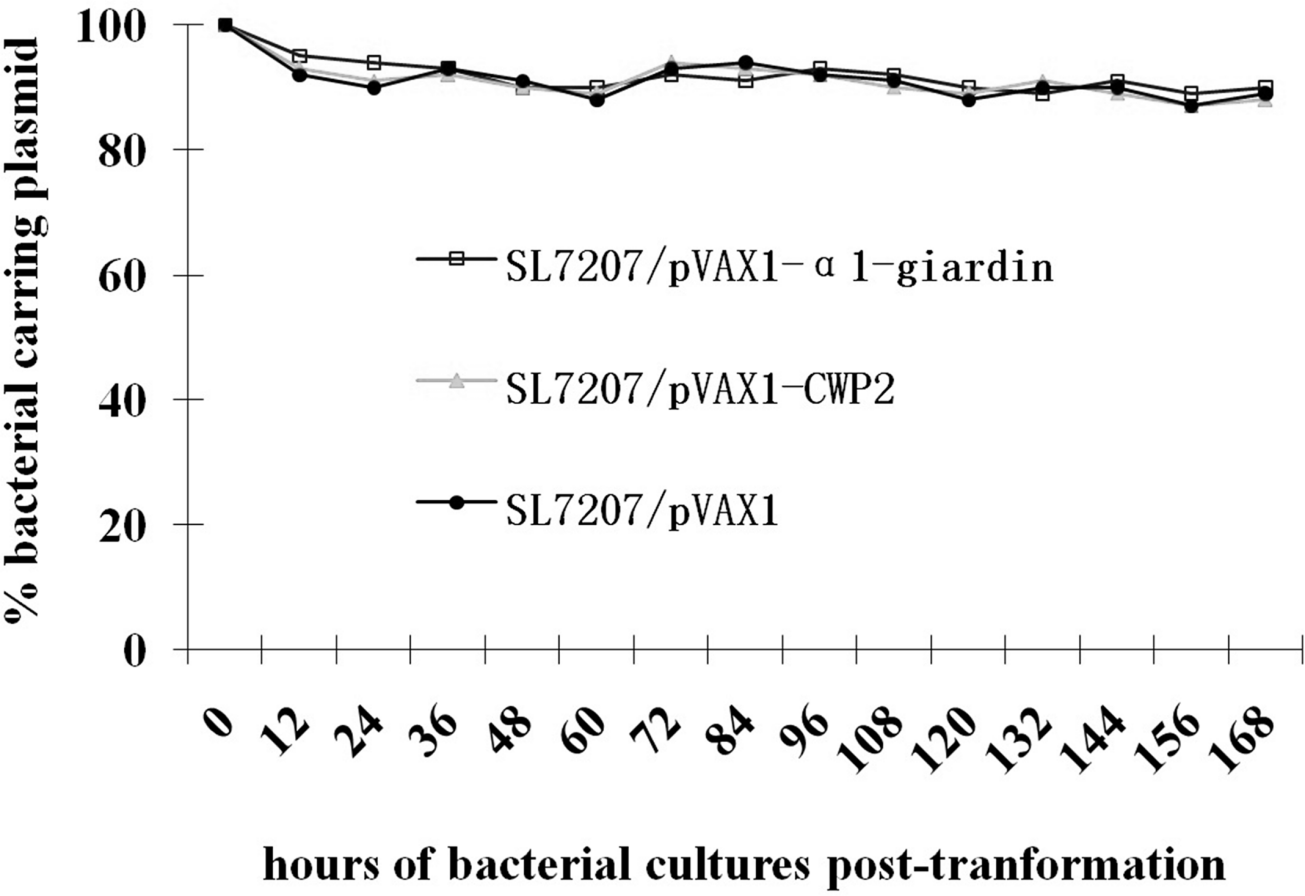
Stability of recombinant plasmid. pVAX1-α1-giardin and pVAX1-CWP2 or pVAX1 alone in transformed SL7207 cultured for 168 hours in antibiotic-free media. The stability was determined by the percentage of bacteria containing the plasmid on kanamycin-LB agar plant compared to the total count on a plate without kanamycin.

**Fig 2:**
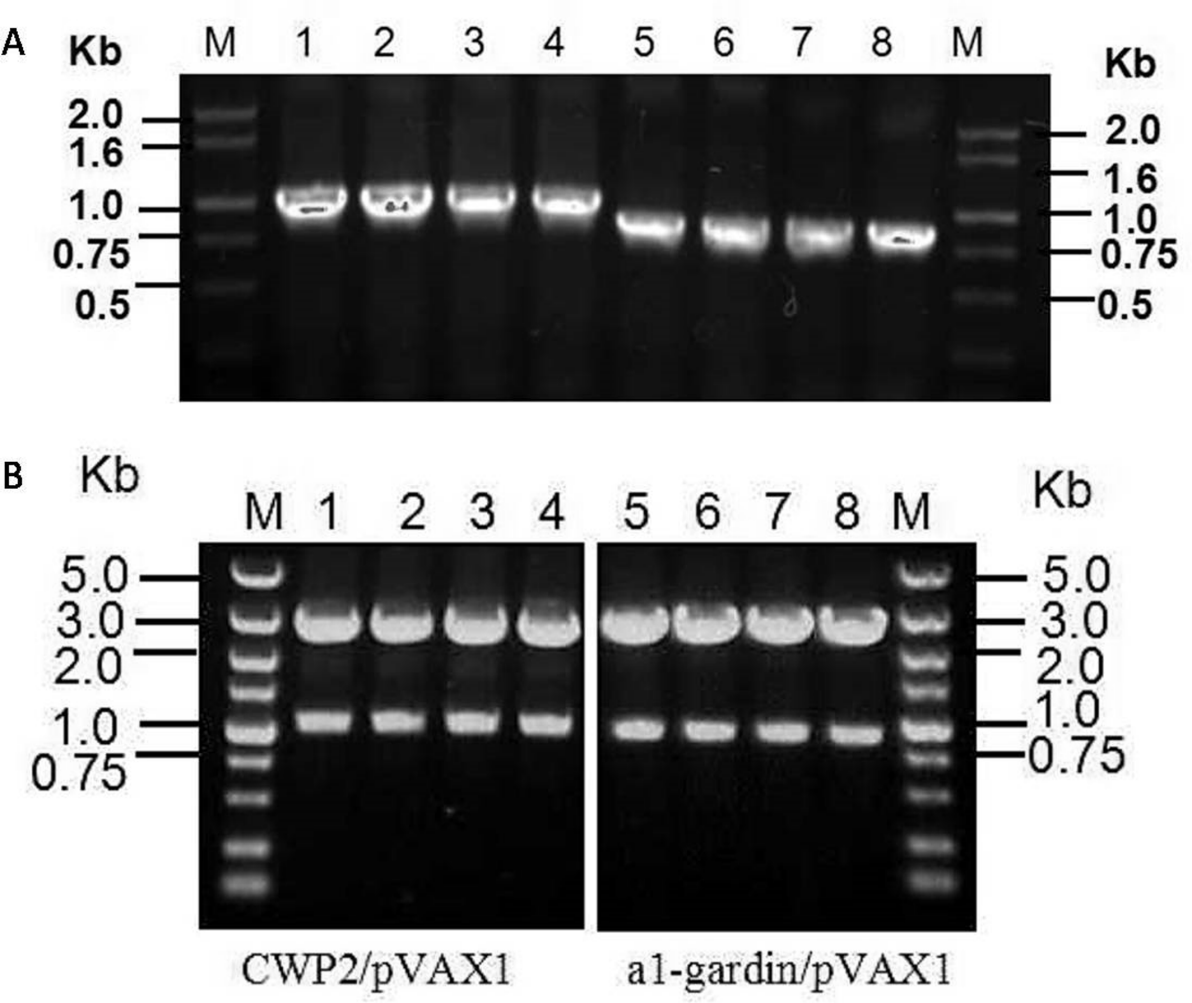
Identification of the α1-giardin and CWP2 insert in isolated plasmids. Toal 4 colonies were randomly picked from plates without kanamycin spread with SL7207/pVAX1-CWP2 (lane 1–4) and SL7207/ pVAX1- α1-giardin (lane 5–8) after being cultured in a kanamycin-free media for 168 hours. The corrected DNA inserts in the extracted plasmid DNAs were confirmed by PCR amplified with vector flanking primers T7 and BGH (A) and by restriction digestion with HimdIII and BamHI (B). M. DNA maker.

The genes encoding α1-giardin and CWP2 were cloned in frame into pVAX1. The correct sequence reading frame was confirmed by double-stranded DNA sequencing using vector flanking primers T7 and BGH. The recombinant pVAX1-α1-giardin and pVAX1-CWP2, as well as the empty vector pVAX1, were transformed into *S*. *typhimurium* SL7207 by electroporation. The stability of the plasmid maintained in the bacteria was determined by comparing the number of survived bacteria on plate containing kanamycin to the total count on plate without kanamycin. After being cultured in LB medium for 7 days without adding kanamycin, 90%, 88% and 89% of bacteria still harbored the plasmid pVAX1-α1-giardin, pVAX1-CWP2 or pVAX1, respectively (Fig 1). Using PCR with primers T7 and BGH and restriction digestion with HindIII and BamHI identified that plasmids extracted from randomly picked four colonies of SL7207 transformed with pVAX1-α1-giardin or pVAX1-CWP2 contained inserted DNAs with correct size (Fig 2).

### The expression of recombinant α1-giardin and CWP2 in MLN of vaccinated mice

**Fig 3:**
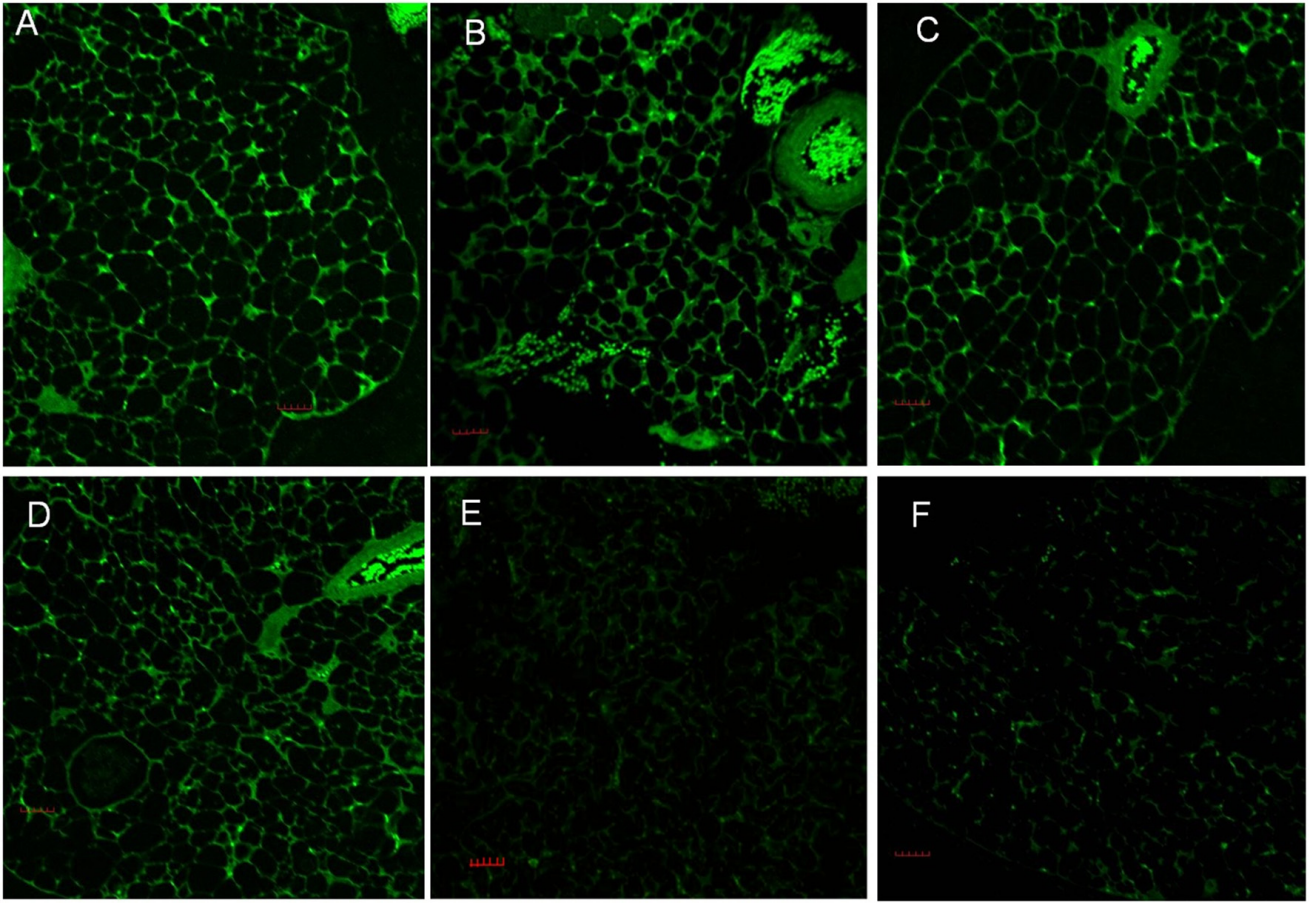
Immunofluorescent detection of α1-giardin or/and CWP2 in MLN by rabbit anti-rα1-giardin and rCWP2-S sera (200×). The expression of rα1-giardin was detected by rabbit anti-rα1-giardin sera in MLN of mouse vaccinated with SL7207/pVAX1-α1-giardin (A). The expression of rCWP2 was detected by rabbit anti-rCWP2-S sera in MLN of mouse vaccinated with SL7207/pVAX1-CWP2 (B). The co-expression of rCWP2 and rα1-giardin in mouse immunized with both SL7207/pVAX1-α1-giardin and SL7207/pVAX1-CWP2 were detected by rabbit anti-rα1-giardin sera (C) and by rabbit anti-rCWP2 sera (D). No obvious fluorescence was detected in the sections of MLNs of mice immunized with SL7207/pVAX1-α1-giardin (E) and with SL7207/pVAX1-CWP2 (F) probed with normal rabbit serum. The bar is 50 μm.

MLNs were collected from mice immunized with *S*. *typhimurium* carrying α1-giardin and CWP2 DNAs and the sections of MLNs were immunostained with anti-α1-giardin or anti-CWP2 rabbit sera. The fluorescent staining revealed that the both recombinant α1-giardin and CWP2 were expressed in MLNs of mice vaccinated with SL7207/pVAX1-α1-giardin and SL7207/pVAX1-CWP2, respectively (Fig 3A and 3B). In the MLN from mice immunized both SL7207/pVAX1-α1-giardin and SL7207/pVAX1-CWP2, expressed both α1-gardin and CWP2 detected by corresponding antisera (Fig 3C and 3D). However, neither α1-gardin nor CWP2 was detected in MLN of mice inoculated with SL7207/pVAX1 only (Fig 3E and 3F). Detection of α1-gardin and CWP2 proteins in MLN suggests that DNAs of α1-gardin and CWP2 are carried to MLN by *Salmonella* bacteria and expressed as recombinant proteins there under the CMV promotor.

### Serological and local antibody responses

**Fig 4:**
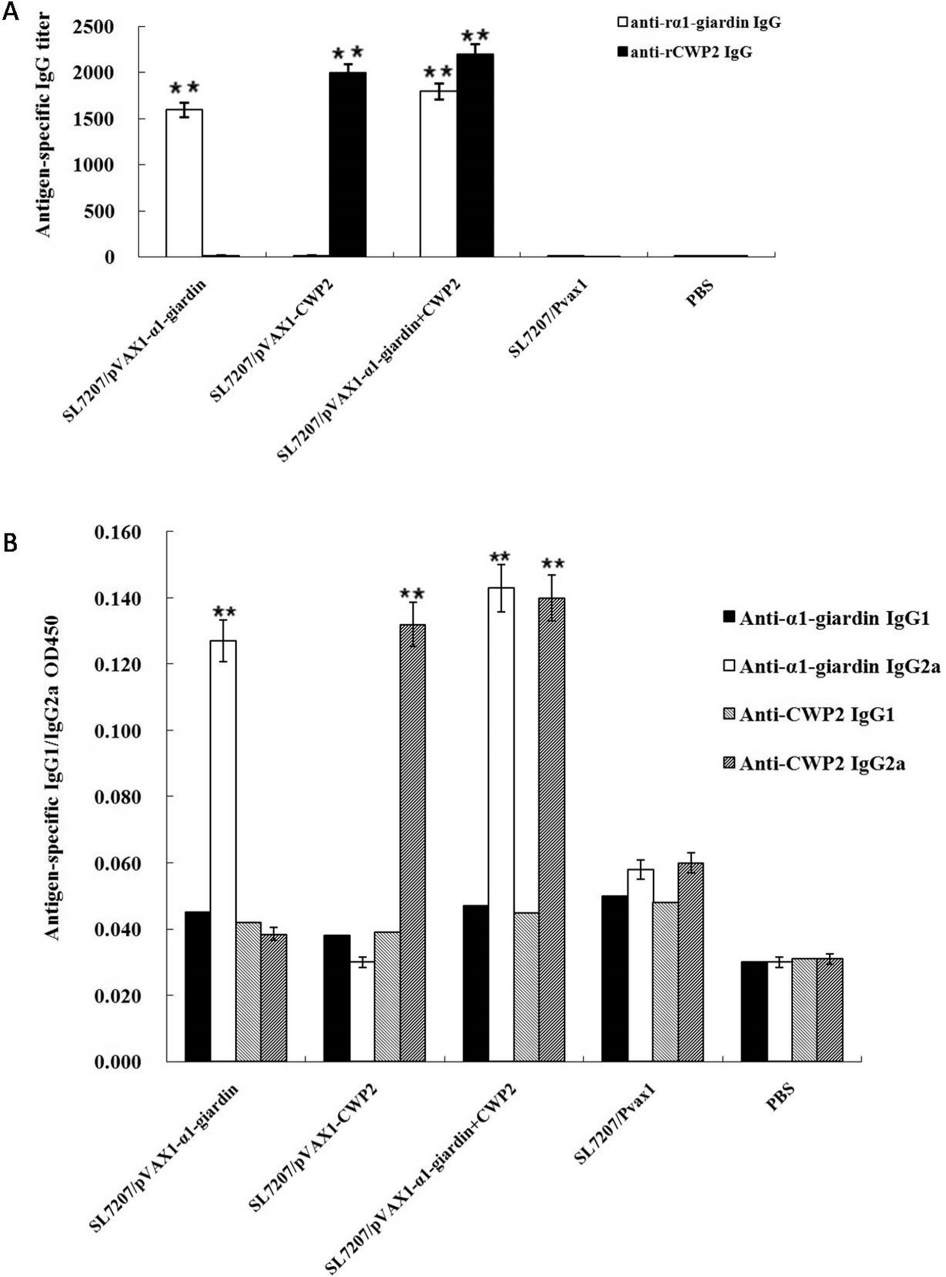
**Mouse IgG (A) and subclass IgG (IgG_1_/IgG_2a_) (B, 1:100 dilution) responses to vaccination with SL7207 carried a1-gardin and CWP2 DNA vaccines measured by ELISA.** Values shown for each group are the mean ± SE of antibody levels (n = 10) (*p<0.05).

The mouse sera were collected from mice vaccinated with SL7207 carrying different DNA vaccines 7 weeks after the last boost vaccination. Mice vaccinated with SL7207 transformed with pVAX1-α1-giardin or pVAX1-CWP2 plasmid produced significantly increased levels of anti-α1-giardin or CWP2-specific IgG antibodies in the sera detected by ELISA compared to non-immunized control mice. However, none of the mice inoculated with SL7207/pVAX1 showed detectable antigen-specific IgG in sera (Fig 4A). The antigen-specific subclass IgG2a was also detected in the sera of immunized mice for both α1-giardin and CWP2, however, there was no detectable IgG1 observed in sera of mice vaccinated with either α1-giardin or CWP2 vaccines compared with PBS or SL7207/pVAX1 control groups (Fig 4B).

**Fig 5:**
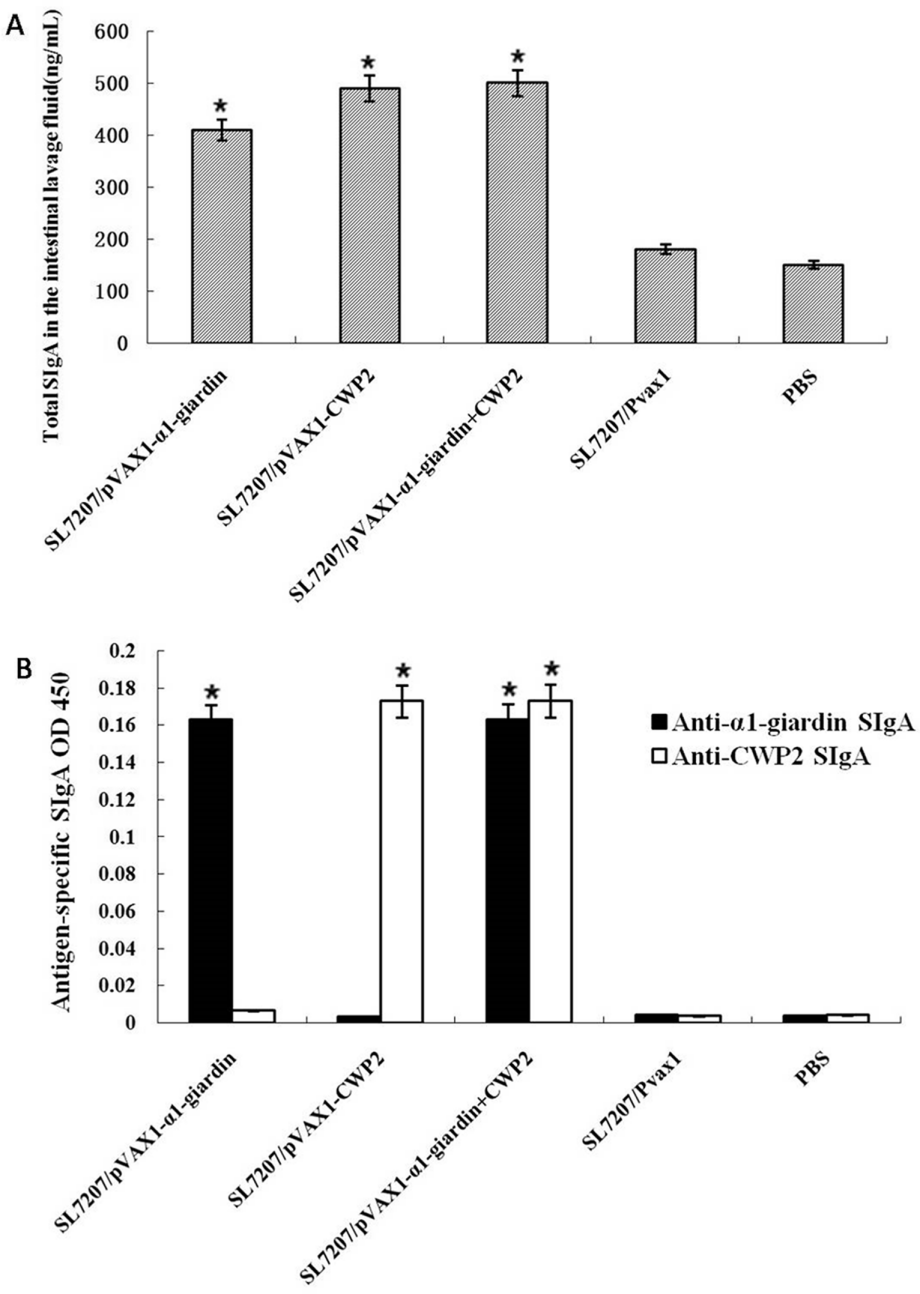
**Intestinal mucosa secreted total IgA (SIgA) (A) and antigen-specific SIgA (B, 1:100 dilution) were measured in the intestinal lavage fluids collected from mice vaccinated with SL7207 carried a1-gardin and CWP2 DNA vaccines measured by ELISA.** Values shown for each group are the mean ± SE of antibody levels (n = 10) (*p<0.05).

Intestinal lavage fluids were collected from vaccinated mice 7 weeks post-vaccination. Intestinal total IgA amounts were significantly increased in mice vaccinated with SL7207/-α1-giardin or pVAX1-CWP2 or both vaccines compared with those inoculated only with vector or PBS (Fig 5A). The antigen-specific IgA levels were also significantly increased in the intestinal mucosa of mice immunized with SL7207/pVAX1-α1-giardin, SL7207/pVAX1-CWP2 or SL7207/-α1-giardin+CWP2. There was no specific mucosal IgA detected in mice inoculated only with vector or PBS (Fig 5B). Values shown for each group are the mean ± SE of antibody levels (n = 10).

### Protective immunity in vaccinated mice against challenge of *G*. *lamblia* trophozoites

**Fig 6:**
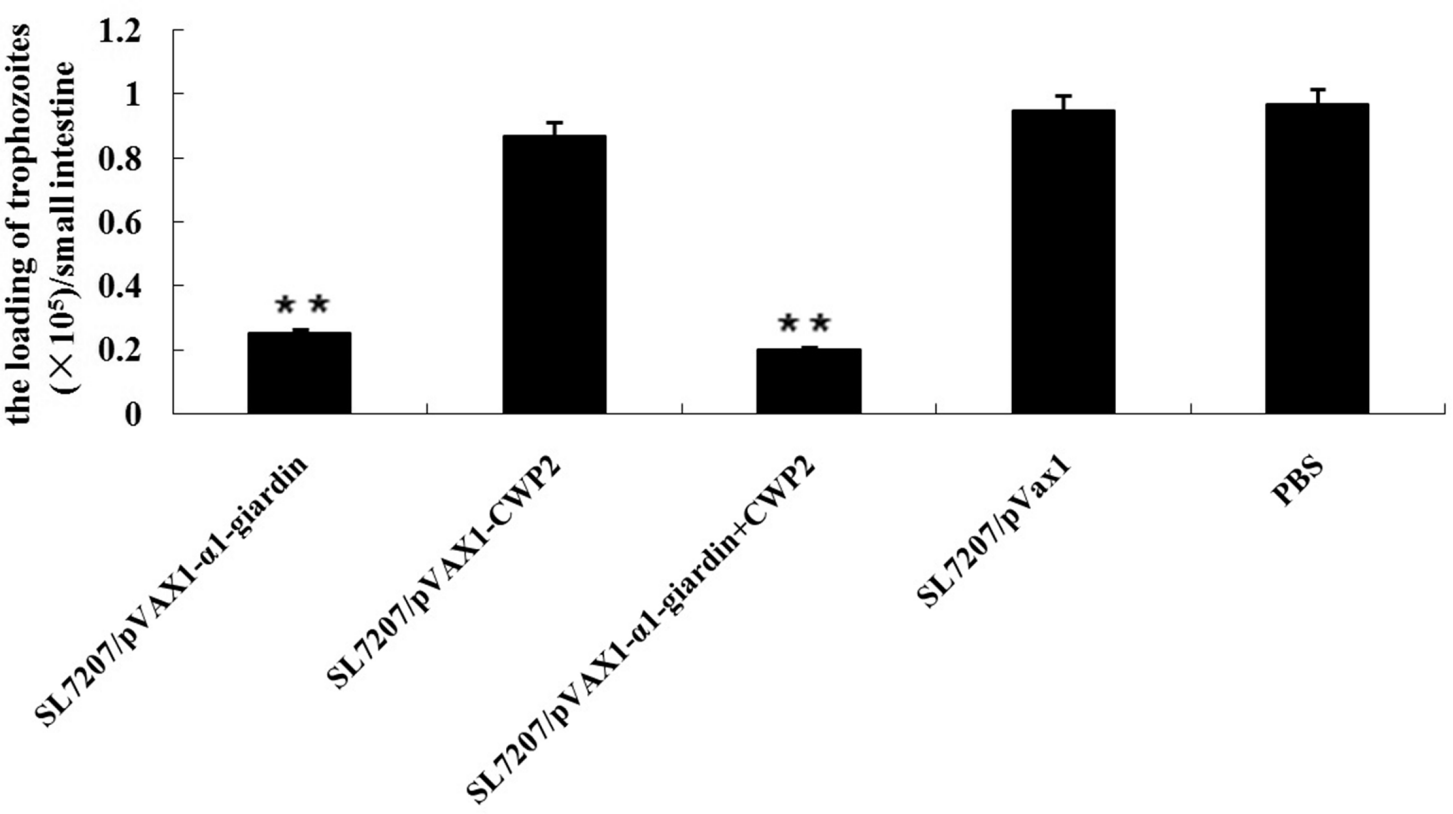
The average trophozoite counts in small intestine of mice of all groups on the fourth day after infection with 1×10^5^ live *Giardia* trophozoites. Values shown for each group are the mean ± SE (n = 10) (*p<0.05,**p<0.01).

**Fig 7:**
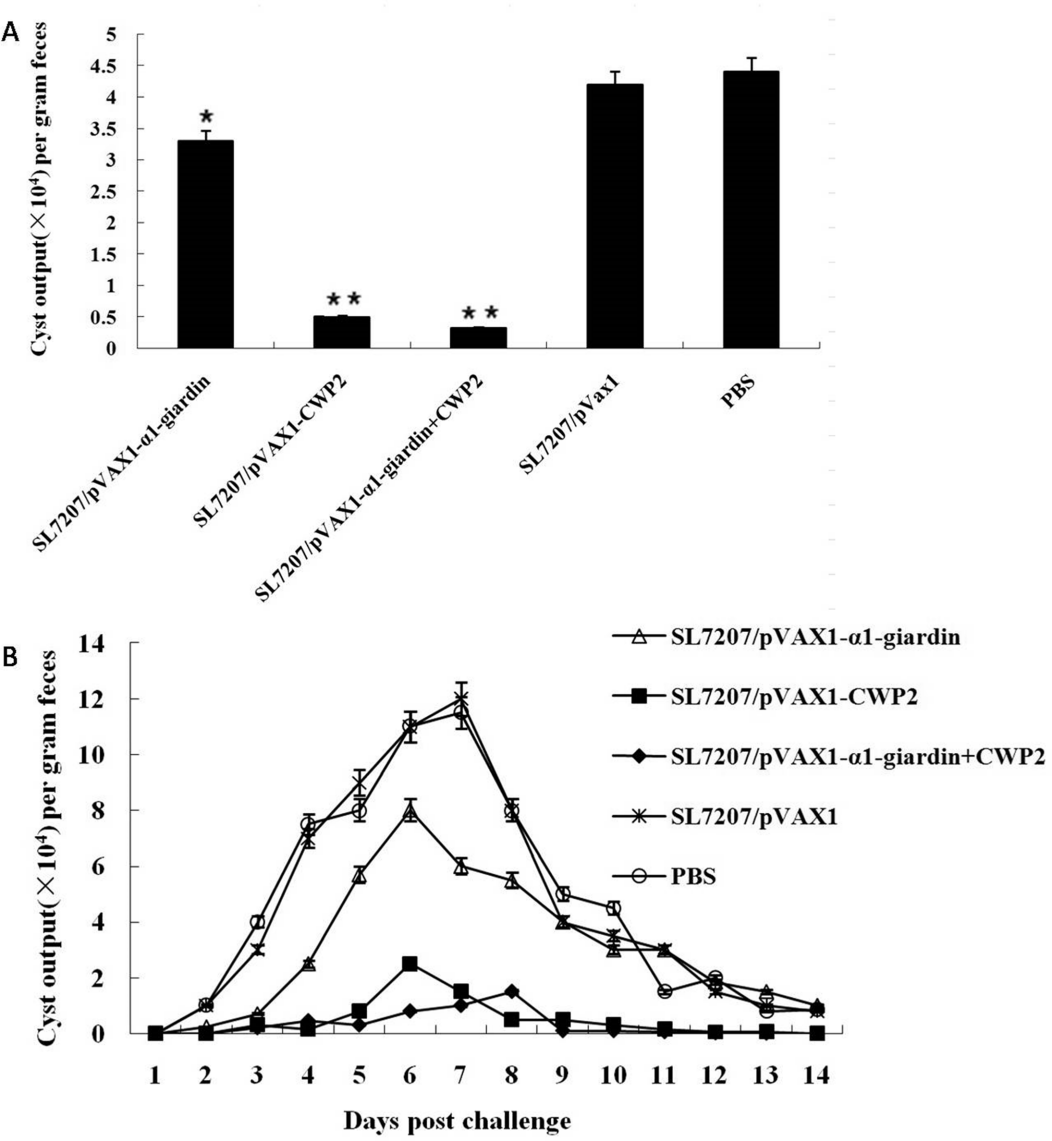
The average cyst output per gram feces of immunized mice over a 2-weeks period after infection. The average number of cysts per gram feces collected during the 2-weeks period was counted for each group (A) (*p<0.05,**p<0.01). Cyst release pattern over a 14-days period post challenge was count for each group (B). Values shown for each group are the mean ± SE (n = 10).

To assess the protective efficacy of oral bivalent DNA vaccines of α1-giardin and CWP2 delivered by attenuated *S*. *typhimurium* against *Giardia* infection, the vaccinated mice were challenged with 1×10^5^ *G*. *lamblia* trophozoites 7 weeks after the last vaccination. The trophozoites in the small intestine of vaccinated mice (10 mice) euthanized on the fourth day after infection were collected and counted and mean output of cysts in the feces of left 10 mice in each group during 2 weeks after infection were counted and calculated. Compared to mice administrated with PBS alone, mice vaccinated with SL7207/pVAX1-α1-giardin obtained 74.2% trophozoite reduction in intestines 4 days post challenge and 28% reduction in the average cyst output in the feces during the two weeks post challenge with both statistical significance (*p*<0.01). Mice vaccinated with SL7207/pVAX1-CWP2 acquired only 10% reduction of trophozoites (*p*>0.05 compared to PBS group), however produced significant reduction of cyst shedding in the feces compared to PBS control (89%, *p*<0.01). When mice were co-vaccinated with SL7207/pVAX1-α1-giardin and SL7207/pVAX1-CWP2, a total of 79% reduction of trophozoite load in intestines and 93% reduction of cyst shedding in feces were obtained compared to the PBS control group with statistical significance (*p*<0.01) (Figs 6 and 7A, Table 1).

**Table 1:** Protection induced by vaccination with attenuated *S*. *typhimurium* SL7207 delivered α1-giardin and CWP2 DNA vaccines in mice.

Group	Trophozoite reduction		Cyst reduction	
	Trophozoite(mean±SD)	Reduction	Cyst (mean±SD)	Reduction
SL7207/pVAX1-α1-giardin	0.25 ± 0.10×10^5^	74.2%**	3.20 ± 0.2×10^4^	28%*
SL7207/pVAX1-CWP2	0.87 ± 0.15×10^5^	10.0%	0.50 ± 0.08×10^4^	89%**
SL7207/pVAX1-α1-giardin/CWP2	0.20 ± 0.13×10^5^	79.0%**	0.32 ± 0.03×10^4^	93%**
SL7207/pVAX1	0.95±0.11×10^5^	0.2%	4.27 ± 0.2×10^4^	0.30%
PBS	0.97±0.17×10^5^	**-**	4.40 ± 0.2×10^4^	**-**

* *P*<0.05

**P<0.01

Furthermore, a time course analysis demonstrated that the protective effects of the oral vaccination with both SL7207/pVAX1-α1-giardin and SL7207/pVAX1-CWP2 vaccines were sustained throughout the acute infection challenge in terms of the cyst shedding. Non-immunized mice (PBS) receiving a *Giardia* infection had a latent phase lasting for 1–2 days followed by an acute phase. By day 14, cysts still could be detected in the feces. Mice receiving plain SL7207/pVAX1 had a pattern of cyst release similar to that of PBS control animals. Oral vaccination with SL7207/pVAX1-CWP2 or combined SL7207/pVAX1-α1-giardin+CWP2 significantly reduced the cyst shedding during the whole 2-weeks period. Mice vaccinated with SL7207/pVAX1-α1-giardin only elicited partial reduction of cyst as shown in Fig 7B.

## Discussion

*Giardia* has a relatively simple life cycle that consists of two different stages including cyst and trophozoite. The trophozoite colonizes the host intestine and the cyst is resistant to environmental conditions, each plays a relatively independent role in *Giardia* colonization/infection and transmission, respectively. Importantly, the expression and composition of antigens are quite different between these two stages of the parasitic protozoan [20]. Therefore, any vaccine using antigen targeting trophozoite or cyst only can not play a preventive role in the infection and the spread of *Giardia*. It will be greatly beneficial to develop a vaccine targeting both trophozoite and cyst to prevent the infection and to block the transmission of giardiasis at the same time.

In the present study, we demonstrated that oral administration of bivalent DNA vaccines targeting trophozoite-specific antigen α1-giardin and cyst-specific CWP2 delivered by attenuated *S*. *typhimurium* SL7027 stimulated both systemic and mucosal immunity against *Giardia* trophozoites and cysts. This immunization induced not only strong local mucosal IgA secretion but also mixed systemic immune responses in mice against *Giardia* parasite. A lot of evidences reveal that secretory IgA (SIgA) plays an important role in mucosal defense and in preventing enteric pathogens from invading surface epithelial layer of the intestine [32]. In our study, oral vaccination with α1-giardin and CWP2 DNAs indeed induced the production and secretion of specific intestinal mucosal IgA, which correlated with the significantly reduced trophozoites and cysts in feces of mice after being challenged with *Giardia* trophozoites. Serum antibody responses showed that the live attenuated *Salmonella* delivered α1-giardin and CWP2 vaccines stimulated an increased level of specific IgG with IgG2a as predominant IgG subclass response. There was much less or none IgG1 response to both α1-giardin and CWP2, indicating *Salmonella* delivered DNA vaccine induce more Th1 than Th2 response that is consistent with earlier studies using single *Giardia* α1-giardin or CWP2 vaccine delivered by attenuated *Salmonella* [13, 18]. Challenge study with *G*. *lamblia* trophozoites demonstrated that the mice vaccinated with *Salmonella*-delieved trophozoite-specific α1-giardin DNA vaccine elicited 74.2% trophozoite reduction in intestines of vaccinated mice compared to mice given with PBS only, consistent with the findings that mice vaccinated with *Salmonella* delivered α1-giardin DNA produced >80% parasite reduction against *Giardia* trophozoite challenge [18]. Interestingly, mice vaccinated with α1-giardin vaccine also produced 28% reduction in cyst shedding, indicating reduced trophozoite results in the less cyst formed in the feces of vaccinated mice. Mice vaccinated with *Salmonella* delivered CWP2 DNA produced 89% reduction in cysts shed in feces of vaccinated mice, that is higher than the finding of Abdul-Wahid and Faubert with 60% reduction against cyst shedding [13]. As expected, mice immunized with CWP2 DNA vaccine did not induce significant reduction in trophozoites compared with PBS control since CWP2 is exclusively expressed in stage of cyst. Significantly, the mice vaccinated with bivalent α1-giardin and CWP2 DNA vaccines delivered by attenuated *Salmonella* produced higher reduction not only against trophozoite loading (79%), but also against encystation of the protozoan (93%) in feces of vaccinated mice. The results demonstrate that bivalent vaccines targeting both trophozoite and cyst antigens can reduce trophozoites in infected mice and may block the transmission of *G*. *lamblia* at the same time because of the reduced cysts. Together, these findings suggest that induction of Th1-type immune responses by *Salmonella* delivered bivalent DNA vaccines of α1-giardin and CWP2 may associate the reduced trophozites and cysts in *G*. *lamblia* infected mice.

These immune responses and the reduced parasite are supported by the observation of recombinant α1-giardin and CWP2 proteins expressed in MLN by immunofluorescent staining with specific antisera. The bacterial strain of *Salmonella typhimurium* SL7027 strain used in this study was attenuated by deleting an aroA gene in the aromatic amino acid biosynthetic pathway that attributes to the pathogenesis [28]. There was no clinical aberrations or body weight reduction in the mice receiving the attenuated *Salmonella* observed during the 14-weeks experimental period in our study, indicating the attenuated *S*.*Typhimurium* is safe for being used as a delivering vector for DNA vaccines in experimental animal.

All together in this study, oral immunization with DNAs encoding for both *Giardia*-specific antigen α1-giardin and CWP2 delivered via attenuated *S*. *typhimurium* elicited a significant reduction in both trophozoites and cysts in feces of mice after being challenged with *Giardia lamblia* trophozoites. This reduction is associated with the strong local mucosal IgA secretion and the IgG2a dominant systemic immune responses in vaccinated mice. This vaccination strategy using multiple antigens from different stages of parasite life cycle to construct a multivalent vaccine can be applicable to other enteric pathogens which have a similar life cycle as *G*. *lamblia*.

## Conclusions

The results demonstrate that bivalent vaccines targeting α1-giardin and CWP2 can protect mice against the colonization of *Giardia* trophozoite and block the transformation of cyst in host at the same time. This parasite reduction is associated with the strong local mucosal IgA secretion and the IgG2a-dominant systemic immune responses in vaccinated mice. The results indicate the bivalent DNA vaccines targeting both trophozoite and cyst antigens could be used to prevent *Giardia* infection but also block the transmission of giardiasis.
